# Prevalence of Fetal Inflammatory Response Syndrome and Villitis of Unknown Etiology in the Placenta of Saudi Women and Their Association with Baby Sex

**DOI:** 10.3390/life14010079

**Published:** 2024-01-02

**Authors:** Waleed Aldahmash, Khaldoon Aljerian, Saleh Alwasel

**Affiliations:** 1 Zoology Department, College of Science, King Saud University, Riyadh 11451, Saudi Arabia; salwasel@ksu.edu.sa; 2 Pathology Department, College of Medicine, King Saud University, Riyadh 11451, Saudi Arabia

**Keywords:** inflammatory response syndrome, VUE, chronic deciduitis, CD8, CD68, placenta, gender

## Abstract

Long-term health consequences are influenced by circumstances that occur during pregnancy. The convergence of the maternal and fetal circulations occurs in the placenta, which is the first organ to develop. Placental pathology provides an accurate diagnosis of amniotic sac inflammation, and pathological alterations in preterm placentas provide evidence for the causes of numerous perinatal pathologies, including spontaneous preterm births. This retrospective study aimed to re-examine placentas regarded as normal by the Obstetrics and Gynecology Department at our institution. Thirty-seven male and forty-seven female placentas were collected following full-term delivery, and the grading and staging of any evident inflammatory responses were evaluated and correlated with the babies’ sex. Full-thickness placental samples that were considered normal and not sent to the histopathology department were obtained from the central and marginal regions of placental discs. Morphological examination of the fresh placenta was conducted, and fetal and maternal inflammatory response syndromes were assessed. In addition, placental villitis of unknown etiology (VUE) and chronic deciduitis were evaluated. Immunohistochemistry was performed to evaluate the patterns of inflammation in the placenta using anti-CD8 and anti-CD68 antibodies. The correlation between silent pathologies and clinical complications or the development of fetal inflammatory response syndrome was measured. In this study, 17 (20%) maternal and 10 (12%) fetal samples showed inflammatory responses. The frequencies of chronic deciduitis and VUE were higher among pregnant Saudi women than previously reported, probably because fetal inflammatory response syndrome goes unnoticed in Saudi Arabia. In addition, the prevalence of fetal and maternal inflammatory responses was higher in the placentas of the mothers of males than in those of females, suggesting that differences occur in the inflammatory response in the placenta depending on the sex of the newborn. Grading placental inflammation (in cases of VUE) typically predicts the degree of maternal anti-fetal cellular rejection; therefore, increasing the number of placental samples sent for microscopic inspection may be preferable because of their significance in identifying the causes of chronic disorders.

## 1. Introduction

Long-term health consequences are influenced by circumstances during pregnancy. The convergence of maternal and fetal circulations occurs in the placenta, which is the first organ to develop [1,2,3,4]. Placental pathology is relatively accurate in diagnosing intraamniotic inflammation [5], and pathological changes in preterm placentas, including chronic chorioamnionitis and deciduitis, may be the cause of spontaneous preterm births and several perinatal pathologies, such as neonatal sepsis, asphyxia, bronchopulmonary dysplasia, and periventricular leukomalacia, in preterm infants [6]. Numerous causes of spontaneous preterm birth require a comprehensive understanding of the etiology, intensity, duration, characteristics, and sites of histological placental inflammation as predictors and determinants of the antenatal environment [7]. Microorganisms in the amniotic fluid have been identified in cases of clinical chorioamnionitis at term [8]. Inflammation and microbial detection in the placenta can also predict birthing complications and explain adverse outcomes [9]. Maternal hyperthermia, leukocytosis, tachycardia, uterine tenderness, and the preterm rupture of membranes are the fundamental signs of clinical chorioamnionitis, which are less frequently observed than in histological chorioamnionitis [10,11].

In cases of intraamniotic infection or inflammation, the source of neutrophils in the amniotic fluid can originate from fetal or maternal origins or a mixture of both. This phenomenon suggests that both the fetus and the mother participate in host defense mechanisms against intraamniotic infection [12]. While acute chorioamnionitis represents a maternal host response, funisitis and chorionic vasculitis signify fetal inflammatory responses. Notably, funisitis and chorionic vasculitis are hallmarks of fetal inflammatory response syndrome and placental disease [13].

Inflammatory changes in the villous placenta, in which maternal T cells infiltrate the chorionic villi by inducing native T-cell chemokines, could be idiopathic. This condition is known as villitis of unknown etiology (VUE). VUE with stem villous obliteration poses a risk of neonatal neurodevelopmental abnormalities a few months after birth, which can manifest near the age of 2 years [1,14]. Chronic chorioamnionitis is a predisposing factor for VUE, and chronic deciduitis may develop when chronic inflammatory infiltrates are present in the basal plate of the placenta [15,16,17]. Toll-like receptors (sTLR2) are a component of the amniotic fluid innate immune system and participate in regulating the inflammatory response to microbial pathogens [18].

Placental chorioamnionitis is associated with ethnic disparities and premature births before 35 weeks [19] and with increased levels of TNF, IL-1, and IL-6 and elevated concentrations of IL-1, IL-6, and IL-8 [20]. In addition, high-grade leukocyte infiltration in placental tissues is associated with elevated levels of TNFα, IL-1β, IL-6, IL-8, p55, p75, IL-1RA, and C-reactive protein in umbilical sera.

Neonatal diseases are associated with advanced chorioamnionitis and elevated levels of both pro- and anti-inflammatory mediators in the umbilical serum [18]. Chorioamnionitis can injure and mature the fetal lung and cause immune nodulation. Postnatal care strategies also change how chorioamnionitis is related to clinical outcomes, such as bronchopulmonary dysplasia [21].

VUE is commonly used to describe the inflammatory infiltration of maternal T cells into fetal chorionic villi, resulting in damaging villous inflammation. T lymphocytes infiltrating the chorionic villi demonstrate immunopositivity for CD3 and CD8. The Hofbauer cells are positive for both CD4 and CD14 expressions, and the macrophages are positive for CD4 and CD68 expressions. Given the lack of diagnostic consensus on the clinical identification of VUE, histological examination reveals various microscopic images that are all subcategorized under the broad spectrum of VUE. Proliferative activity, with areas of necrosis and granulation tissue formation, is visible when chronic villi are affected. These phenotypes are either distal, where terminal or mature intermediate villi are evident, or proximal, when stem villi are involved. The basal type comprises anchored villi that result in chronic deciduitis. Redline classified VUE into low- and high-grade VUE, distinguishing between the involvement of whether ten villi were affected per focus. Low-grade VUE is defined as the presence of inflammation affecting fewer than ten contiguous villi in any one focus; more than one focus is required for diagnosis. High-grade VUE is defined as the presence of multiple foci in more than one section, with at least one exhibiting inflammation that affects more than ten contiguous villi [22].

Chronic deciduitis is diagnosed based on the presence of lymphoplasmacytic inflammation in the decidua or, in the absence of plasma cells, the presence of diffuse and intense (>50/HPF) nonperivascular lymphocytic inflammation [23]. VUE exhibits minimal vasculitis or perivasculitis that may cause fetal vascular destruction or thrombotic occlusions. Certain cytological features and immunohistochemical staining distinguish infectious villitis from VUE. Causative infectious organisms include syphilis, cytomegalovirus, parvovirus B19, and rubella. Due to advances in vaccination, virus-induced villitis is now rare.

This retrospective study aimed to re-examine placentas regarded as normal by the Obstetrics and Gynecology Department at our institution. Grading and staging of any clear inflammatory responses were evaluated according to the sex of newborns. This study also aimed to determine the relationship between inflammatory and pathological features of the placenta.

## 2. Materials and Methods

### 2.1. Study Design

This study was conducted at King Saud University Medical City in Riyadh, Saudi Arabia between January and August 2019. This study focused on placentas that were considered normal by the Obstetrics and Gynecology Department and were not sent to the histopathology department. This study was approved by the institutional review board (IRB: E-17-2729). The informed consent of the women participating in this study included a description of their medical reports and collection of placental tissues.

### 2.2. Placental Samples

Eighty-four full-term placentas were collected immediately after delivery (thirty-seven male and forty-seven female placentas). Placentas from women with chronic diseases, non-Saudi women, and twin deliveries were excluded. During pregnancy follow-up, the TORCH test-positive cases were excluded. In addition, the histological slides excluded etiologies such as viral, fungal, protozoan, bacterial, and acute inflammation (by neutrophils). The measurements of fresh placentas, including weight, length, and width, were recorded for morphological examination. The length, coiling, and diameter of the umbilical cords were also measured.

### 2.3. Histological Study

Five samples were taken from each case: one from the fetal membrane roll, two from the umbilical cord (one from the area near the placental disc and the other from the area near the newborn’s body), and two full-thickness samples of placental disc were obtained and collected from the central and marginal regions. The placental samples were fixed in neutral buffered formalin (10%) and then dehydrated by passing them through an upward series of ethanol. Subsequently, samples were cleared in xylene and embedded in paraffin wax. The blocks were sectioned into 3–5 µm and stained with hematoxylin–eosin stain. The standards suggested by the Amsterdam Placental Workshop Group were used to rate placental inflammation, including maternal and fetal inflammatory responses, VUE, and chronic deciduitis [22].

Different sections of placental tissue were selected for microscopic examination, depending on the extent of inflammation. An Olympus BX63 microscope with a DP80 digital camera connected to cellSens 2.1 Entry imaging software was used for the examination and imaging of placental tissue sections.

### 2.4. Immunohistochemistry Staining

Paraffin wax blocks of each of the studied placentas were evaluated using immunohistochemistry (IHC) with murine monoclonal anti-CD8 (C8/144B—IHC—Prediluted [NBP2-45325] Novus Biologicals, Centennial, CO, USA) and anti-CD68 (PG-M1; [M0876], Dako, Carpinteria, CA, USA; 1:20) to detect inflammatory cells. Blocks were cut to 3 µL in thickness, and the paraffin was removed by passing tissue sections over xylene twice for 10 min each time. The tissue sections were hydrated with a descending series of ethanol and immersed in distilled water for 5 min at each stage. Tissue sections were incubated with peroxidase and then with a protein block for 5 min each. The antibodies were diluted according to the manufacturer’s instructions, and the tissue sections were incubated with primary antibody overnight at 4 °C. The primary antibodies were removed, and the tissue sections were washed, followed by incubation with secondary antibodies for 30 min at room temperature. The procedures were performed according to the manufacturer’s instructions. Hotspots were assessed at 400× magnification by two pathologists and using histomorphometry. The morphological evaluation of IHC expression was conducted using ImageJ software (ImageJ bundled with 64-bit Java 8) to computationally measure the area fraction.

### 2.5. Statistical Analysis

Data were presented as means ± standard deviation and percentages. Descriptive analysis, independent sample *t*-tests, Pearson’s chi-square test, and Pi correlation coefficient were performed using SPSS version 25 (IBM Inc., Armonk, NY, USA). Differences were considered statistically significant at *p* < 0.05.

## 3. Results

### 3.1. Anthropometric of Population Study and Placental Inflammation

Characteristics of the pregnant women who participated in this study and their offspring are listed in Table 1. On average, the pregnant women were 30 years old, weighed 79 kg, stood 158 cm high, and had a body mass index of 32 kg/m^2^. Babies in this study measured 3179 g in weight and 49.7 cm in length. The average placental weight was 446 g.

**Figure 1:**
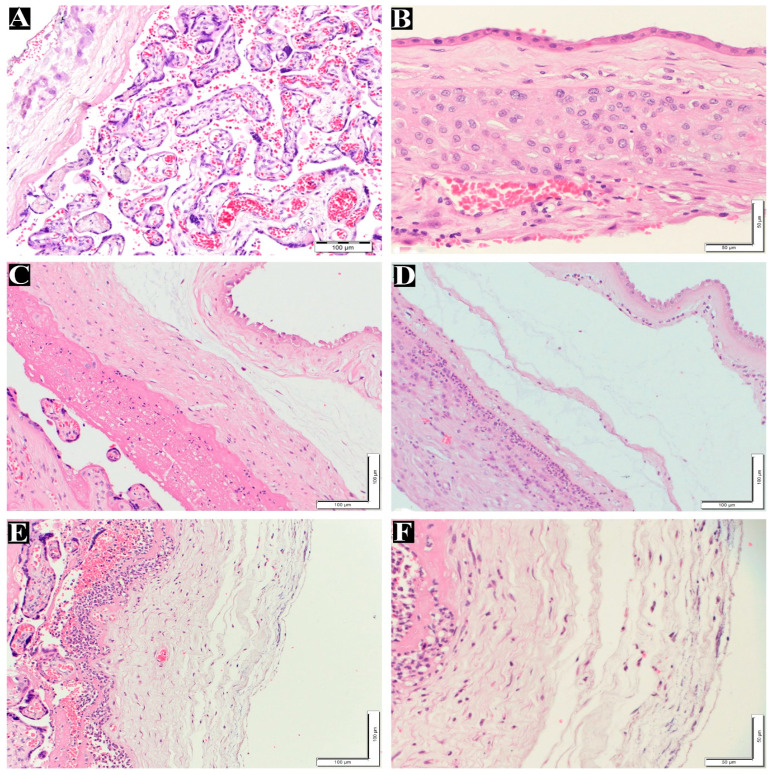
Maternal inflammatory response stages and grades in the placentas. (**A**) Normal villi and decidua layer without inflammatory cells (200×). (**B**) Normal membrane without inflammatory cells in the chorion and amnion layers (400×). (**C**) Maternal inflammatory response in Stage 1 (chorionitis), Grade 1 200×. (**D**) Maternal inflammatory response in Stage 2, Grade 2 (200×). (**E**) Maternal inflammatory response in Stage 3, Grade 2 (200×). (**F**) High magnification for picture. (**E**) Maternal inflammatory response in Stage 3, Grade 2 (400×). All the above tissues were stained with H&E.

**Figure 2:**
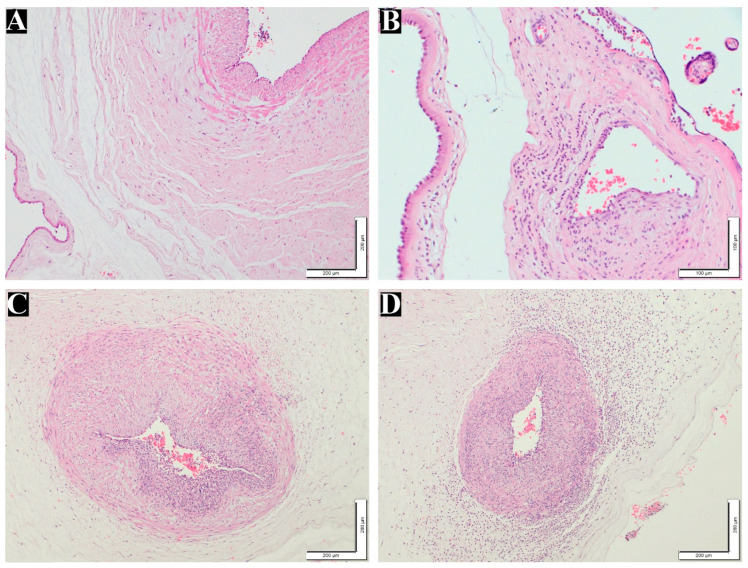
Fetal inflammatory response stages and grades in the placentas. (**A**) Normal umbilical artery (100×). (**B**) Fetal inflammatory response in Stage 1, Grade 1 (200×). (**C**) Fetal inflammatory response in Stage 2, Grade 2 (100×). (**D**) Fetal inflammatory response in Stage 3, Grade 2 (100×). All the above tissues were stained with H&E.

After examining the placental samples using light microscopy and IHC, representative photomicrographs were captured for histomorphometric analysis, and the clinical and histological findings were correlated. The maternal inflammatory response was positive in 17 (20.2%) of the 84 patients. The staging of the positive cases was eight (9.5%), seven (8.3%), and two (2.4%) in Stages 1, 2, and 3, respectively. Moreover, eight (9.6%) and nine (10.7%) cases were in Grades 1 and 2, respectively. For the fetal inflammatory response, only 10 (11.9%) cases were positive among 84 patients. Of these positive responses, 10 (11.9%) cases were in Stage 1, with eight and two cases in Grades 1 and 2, respectively (Table 2). No cases were detected at any of the other stages. Figure 1A–F show normal and maternal inflammatory responses in the placenta, and fetal inflammatory responses are illustrated in Figure 2B–D.

**Figure 3:**
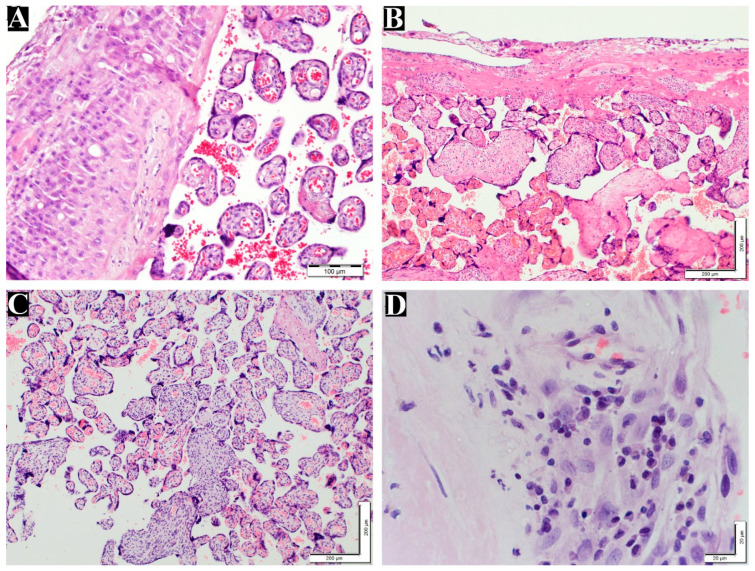
Grade of VUE and chronic deciduitis in placentas. (**A**) Normal chorionic villi and decidua layer (200×). (**B**) Low-grade VUE (100×). (**C**) High-grade VUE (100×). (**D**) Chronic deciduitis with plasma cells (600×). All the above tissues were stained with H&E.

**Figure 4:**
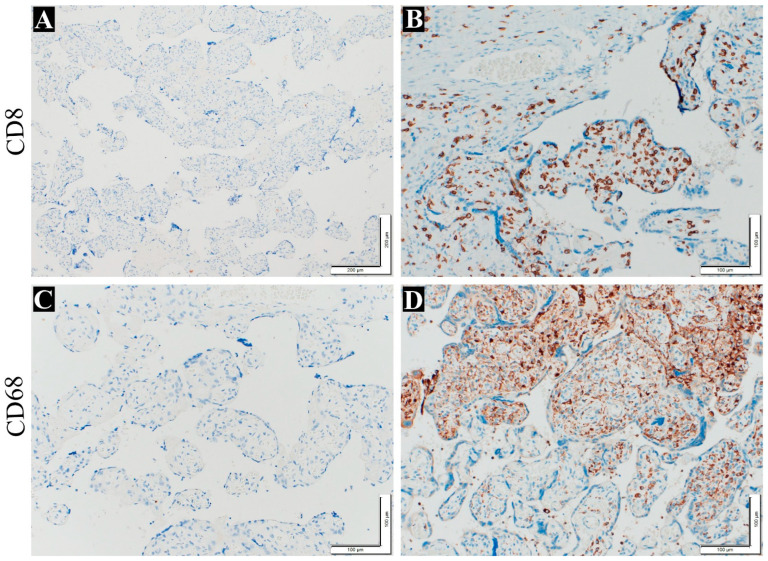
IHC staining to detect VUE in placentas. (**A**) Negative control slide without anti-CD8 (100×). (**B**) T cell stain with anti-CD8 (200×). (**C**) Negative control slide without anti-CD68 (200×). (**D**) Macrophage cell stain with anti CD68 (200×).

Table 3 shows the numbers and percentages of the VUE and chronic deciduitis cases diagnosed in the maternal placentas. The VUE and decidua data are shown in Figure 3. IHC was performed to confirm the presence of inflammatory cells (plasma cells and T lymphocytes) in the chorionic villi, and the results were positive for antibodies against CD8 and CD86 (Figure 4).

### 3.2. Anthropometric of Population Study and Placental Inflammation According to the Sex of the Babies

Differences in the study population were examined based on the sex of the newborns. Of the 84 pregnant women who participated in this study, 37 gave birth to males, and 47 gave birth to females. The findings revealed no significant variations in the measurements of mothers, newborns, or placentas between male and female newborns, except for the height of the mothers, where mothers of females were taller than those of males (Table 4).

The prevalence of fetal and maternal inflammatory responses was higher in the placentas of mothers of males than in those of females (Table 5). However, no significant difference was observed in the percentages of VUE or chronic deciduitis in the placentas of mothers of either sex (Table 6). Table 7 compares the population characteristics based on the presence of an inflammatory model in the placenta. The placentas of less obese mothers showed signs of maternal and fetal inflammation. VUE has also been observed in placentas with a lower weight.

Table 8 reveals the correlation between inflammatory features of the placenta, indicating a positive correlation between the maternal and fetal inflammatory responses. The results also indicate a positive correlation between chronic deciduitis and the maternal and fetal inflammatory responses. Moreover, a strong positive correlation is present between maternal and fetal inflammatory responses. The coexistence of chronic chorioamnionitis and VUE was not detected in the positive cases in the current study.

## 4. Discussion

Because mother-to-fetus nutrition occurs via the placenta, successful pregnancy and fetal growth depend on the functionality of the normal placenta and maternal circulation. Maternal growth factors that are required for placental development and their corresponding mechanisms of action have been reviewed by Forbes et al. [24]. In the past decade, research on the developmental causes of health and disease has focused on the placenta [25]. The placenta has recently been considered the “center of the chronic disease universe” [26]. Placental inflammation is a sub-focus in the investigation of the risk of chronic diseases, particularly considering the worldwide obesity pandemic [4]. Numerous factors can cause placental inflammation, including maternal autoimmune diseases, genetic risk factors, obesity, and immune responses to infection [1] by bacteria [27,28,29], viruses [30], and other infectious organisms.

Previous studies have indicated that VUE can appear in 5–15% of placentas [31]. This finding is consistent with the results of the current study. However, the molecular mechanisms underlying the development of VUE remain unclear. Although Perforin-1 and granzyme B (GrzB) have been reported to play pivotal roles in causing cell-mediated immune responses that trigger a cascade of caspases to initiate cytolysis and apoptosis, this molecular evidence is not applicable to pathologists. Other studies suggest that the activation of C5 initiates an inflammatory reaction [32]. Cole et al. [33] concluded that stimulating mucosal-associated invariant T (MAIT) cells by IL-7, IL-12, IL-15, or IL-18 triggers the secretion of interferon-γ, tumor necrosis factor-α, and IL-17. Moreover, MAIT cells mediate cytotoxic effects via GrzB and perforin. The degradation of GrzB reduces the efficacy of NK cell-mediated lysis, thus minimizing sensitivity and enhancing immune escape ability [34]. Histologically, chronic chorioamnionitis is characterized by inflammatory infiltrates extending into the chorioamniotic membranes or chorionic plate. The immunoreactivity of these cells is typically irregular or diffuse immunopositivity for maternal CD8+ T cells. Trophoblast damage caused by CD8+ T cells in the form of apoptosis was demonstrated using double immunofluorescence staining with antibodies against CD8+ lymphocytes and M30. The presence of lymphocytes and plasma cells in the basal plate of the placenta suggests chronic deciduitis. This inflammatory cell population is thought to migrate to the basal plate because of a microbial or immunological etiopathogenesis. In our study, the frequencies of chronic deciduitis and VUE were higher than that previously reported in the placental pathology literature [35].

The grading of placental inflammation typically predicts the degree of maternal anti-fetal cellular rejection. Screening for anti-fetal antibody-mediated rejection by identifying maternal serum antibodies against fetal HLA and determining whether they are specific to the fetus in the index pregnancy are possible [36]; however, we did not examine these in the current study. Although immunohistochemical analysis using CD68 is the gold standard, CD163, perforin, GrzB, granzyme K, and C5b-9 appear to be important proteins in placental pathology.

According to the findings of this study, fetal and maternal inflammatory responses were more frequent in the placentas of mothers of males than in those of females. Similar studies have demonstrated an increased cytokine response in the plasma of male infants compared to that of females at birth [37,38]. Cytokine antagonists are upregulated to maximize the labor-induced inhibition of cytokine production that contributes to parturition [20]. Cytokines participate in placental paracrine or autocrine regulatory networks during the second and third trimesters to protect the fetus from pathological organisms. They also contribute to fetal expulsion via uterine contractions, membrane rupture, and cervical dilation [39]. Although the extraplacental membranes change during normal-term parturition, labor-associated changes in the villous placenta are significant [39]. IL-6 is a diagnostic marker of intra-amniotic inflammation that predicts the risk of impending preterm delivery [40]. The increased incidence of pregnancy complications in male fetuses compared to female fetuses, including spontaneous abortions, preterm birth, and preterm premature rupture of membranes [41,42,43], may be explained by the sex-specific differences in the inflammatory response discovered in the placenta in this study.

Maternal and fetal inflammatory responses tend to appear in the placentas of pregnant women with low body mass, and VUE tends to occur in low-weight placentas, as shown by the results of this study. Goldstein et al. [1] indicated that pregnant women with a high BMI tend to have chronic inflammation. Moreover, other earlier investigations have reported that fetal growth restriction, preterm delivery, and low birth weight are all linked to increased placental inflammation [17,31,44]. These results may be due to the differences in the lifestyles of the study population.

The findings of this study revealed a correlation among the inflammatory aspects of the placenta and agrees with previous studies [15,16,29].

## 5. Conclusions

In cases of VUE, the grading of placental inflammation typically predicts the degree of maternal anti-fetal cellular rejection. Screening for anti-fetal antibody-mediated rejection by identifying maternal serum antibodies against fetal HLA and determining whether they are specific to the fetus during pregnancy are possible. The placental inflammatory response differs according to the sex of the newborn, which may explain certain complications that occur in mothers of males. Because placental pathologies were undiagnosed, several cases of fetal inflammatory response syndrome were unnoticed in this study’s population. Therefore, expanding the types of placental samples sent for microscopic examination and studying their relationship with maternal and newborn measurements are important to discovering the causes of chronic diseases.

## Figures and Tables

**Table 1:** Anthropometrics of the study population.

		Mean	Std. Deviation
**Mothers**	Age (year)	30.4	5.62
	Height (cm)	157.96	4.96
	Weight (kg)	79.47	14.57
	Parity	1.66	1.72
	Body mass index (kg/m^2^)	31.76	5.57
**Babies**	Gestational age (week)	38.6	1.45
	Birth weight (g)	3179	442
	Length (cm)	49.7	2.2
**Placenta**	Weight (g)	445.6	86.5
	Length (cm)	19.7	3.2
	Width (cm)	16.2	1.8
**Umbilical Cord**	Length (cm)	53.1	11.5
	Diameter (cm)	1.2	0.2
	No. Coiling	3.65	0.96

**Table 2:** Fetal and maternal inflammatory response changes in the placenta.

			No. (%)
**Maternal inflammatory response**	Positive		17 (20.2)
	*Stage*	1	8 (9.5)
		2	7 (8.3)
		3	2 (2.4)
	*Grade*	1	8 (9.6)
		2	9 (10.7)
**Fetal inflammatory response**	Positive		10 (11.9)
	*Stage*	1	10 (11.9)
		2	0 (0)
		3	0 (0)
	*Grade*	1	8 (9.5)
		2	2 (2.4)

**Table 3:** VUE and chronic deciduitis in the placentas.

			No. (%)
**VUE**	Positive		8 (9.5)
	*Grade*	Low	4 (4.8)
		High	4 (4.8)
**Chronic deciduitis**	Positive		55 (65.5)
		Lymphocytes > 50	31 (36.9)
		Plasma cells	24 (28.6)

**Table 4:** Comparison of anthropometrics of the study population between male and female babies.

		Male (n = 37)	Female (n = 47)	
		Mean ± SD	Mean ± SD	*p* Value
**Mothers**	Age (years)	29.3 ± 5.01	31.2 ± 5.96	0.1
	Height (cm)	156.4 ± 3.8	159.1 ± 5.4	0.02
	Weight (kg)	76 ± 13	82 ± 15.2	0.06
	Parity	1.25 ± 1.5	1.98 ± 1.8	0.055
	Body mass index (kg/m^2^)	30.8 ± 5.3	32.5 ± 5.7	0.2
**Babies**	Gestational age (week)	38.5 ± 1.7	38.7 ± 1.3	0.5
	Birth weight (g)	3152 ± 444	3199 ± 444	0.6
	Length (cm)	49.9 ± 2.3	49.5 ± 2.2	0.4
	Head Cir. (cm)	34 ± 1.3	34.3 ± 1.4	0.2
	Chest Cir. (cm)	33.7 ± 2.7	33 ± 2	0.2
	Thigh Cir. (cm)	16.4 ± 1.8	16 ± 1.7	0.3
**Placenta**	Weight (g)	428.5 ± 78	458.7 ± 90	0.1
	Length (cm)	19.4 ± 3.2	19.9 ± 3.3	0.5
	Width (cm)	16 ± 1.7	16.4 ± 1.8	0.3
**Umbilical Cord**	Length (cm)	54.7 ± 10.7	51.8 ± 12	0.2
	Diameter (cm)	1.2 ± 0.2	1.1 ± 0.2	0.1
	No. Coiling	3.4 ± 0.9	3.8 ± 1	0.1

**Table 5:** Fetal and maternal inflammation responses in the placenta according to male and female babies.

			Male (n = 37)	Female (n = 47)	*p* Value
			N (%)	N (%)	
**Maternal Inflammatory Response**	Positive		12 (32.4)	5 (10.6)	0.01
	*Stage*	1	7 (18.9)	1 (2.1)	
		2	3 (8.1)	4 (8.5)	
		3	2 (5.4)	0 (0)	
	*Grade*	1	5 (13.5)	3 (6.4)	
		2	7 (18.9)	2 (4.3)	
**Fetal Inflammatory Response**	Positive		8 (21.6)	2 (4.3)	0.01
	*Stage*	1	8 (21.6)	2(4.3)	
		2	0	0	
		3	0	0	
	*Grade*	1	7 (18.9)	1 (2.1)	
		2	1 (2.7)	1 (2.1)	

**Table 6:** VUE and chronic deciduitis in the placentas according to the sex of babies.

			Male (n = 37)	Female (n = 47)	*p* Value
			N (%)	N (%)	
**VUE**	Positive		6 (16.2)	2 (4.2)	0.06
	*Grade*	Low	3 (8.1)	1 (2.1)	
		High	3 (8.1)	1 (2.1)	
**Chronic Deciduitis**	Positive		21 (56.8)	34 (72.3)	0.3
		Lymphocytes > 50	11 (29.7)	20 (42.6)	
		Plasma cells	10 (27.1)	14 (29.8)	

**Table 7:** Comparison between population anthropometrics based on the appearance of placental inflammatory responses.

	Maternal Inflammation			Fetal Inflammation			VUE			Chronic Deciduitis		
	Negative	Positive	*p* Value	Negative	Positive	*p* Value	Negative	Positive	*p* Value	Negative	Positive	*p* Value
**Mother’s BMI (kg/m^2^)**	32.6	28.3	*0.01*	32.3	27.6	*0.02*	31.9	30.2	*0.36*	30.5	32.4	*0.21*
**Baby weight (g)**	3135	3351	*0.07*	3170	3248	*0.60*	3188	3095	*0.57*	3224	3155	*0.49*
**Placental weight (g)**	440	469	*0.22*	445	452	*0.81*	452	387	*0.04*	454	441	*0.53*
**Gestational age (week)**	38.5	39.2	*0.09*	38.6	39.2	*0.20*	38.6	39	*0.47*	38.8	38.5	*0.45*

VUE: Villitis of unknown etiology; BMI: Body mass index.

**Table 8:** Correlations between the inflammatory features of the placenta.

	Maternal Inflammatory Response	Fetal Inflammatory Response	VUE
**Fetal Inflammatory Response**	0.73 ***		
**VUE**	0.06	0.01	
**Chronic Deciduitis**	0.26 *	0.27 *	0.06

(*) Correlation is significant at the 0.05 level; (***) Correlation is significant at the 0.01 level.

## Data Availability

The data presented in this study are available in this article.
